# Beyond PrP^res^ Type 1/Type 2 Dichotomy in Creutzfeldt-Jakob Disease

**DOI:** 10.1371/journal.ppat.1000029

**Published:** 2008-03-14

**Authors:** Emmanuelle Uro-Coste, Hervé Cassard, Stéphanie Simon, Séverine Lugan, Jean-Marc Bilheude, Armand Perret-Liaudet, James W. Ironside, Stéphane Haik, Christelle Basset-Leobon, Caroline Lacroux, Katell Peoch', Nathalie Streichenberger, Jan Langeveld, Mark W. Head, Jacques Grassi, Jean-Jacques Hauw, Francois Schelcher, Marie Bernadette Delisle, Olivier Andréoletti

**Affiliations:** 1 INSERM U858, Institut de Médecine Moléculaire de Rangueil and Service d'Anatomie Pathologique et Histologie-Cytologie, C.H.U. Rangueil, Toulouse, France; 2 UMR Institut National de la Recherche Agronomique (INRA)/Ecole Nationale Vétérinaire de Toulouse (ENVT) 1225, Interactions Hôtes Agents Pathogènes, ENVT, Toulouse, France; 3 Commissariat à l'Energie Atomique (CEA), Service de Pharmacologie et d'Immunologie, DRM, CEA/Saclay, Gif sur Yvette, France; 4 Bio-Rad, Research and Development Department, Marnes-la-Coquette, France; 5 Hôpital Neurologique, Services de Neurochimie et de Pathologie, Bron, France; 6 National Creutzfeldt-Jakob Disease Surveillance Unit, Division of Pathology, University of Edinburgh, Western General Hospital, Edinburgh, United Kingdom; 7 INSERM, Equipe Avenir, Maladies à Prions chez l'Homme, Paris, France; 8 Neuropathology Laboratory, Salpêtrière Hospital, AP-HP, Paris, France; 9 Service de Biochimie et Biologie Moléculaire, Hôpital Lariboisière, Paris (Laboratoire associé au CNR “ATNC”) et EA 3621 Faculté de Pharmacie, Paris, France; 10 Central Institute for Animal Disease Control CIDC-Lelystad, Lelystad, The Netherlands; University of Alberta, Canada

## Abstract

Sporadic Creutzfeldt-Jakob disease (sCJD) cases are currently subclassified according to the methionine/valine polymorphism at codon 129 of the *PRNP* gene and the proteinase K (PK) digested abnormal prion protein (PrP^res^) identified on Western blotting (type 1 or type 2). These biochemically distinct PrP^res^ types have been considered to represent potential distinct prion strains. However, since cases of CJD show co-occurrence of type 1 and type 2 PrP^res^ in the brain, the basis of this classification system and its relationship to agent strain are under discussion. Different brain areas from 41 sCJD and 12 iatrogenic CJD (iCJD) cases were investigated, using Western blotting for PrP^res^ and two other biochemical assays reflecting the behaviour of the disease-associated form of the prion protein (PrP^Sc^) under variable PK digestion conditions. In 30% of cases, both type 1 and type 2 PrP^res^ were identified. Despite this, the other two biochemical assays found that PrP^Sc^ from an individual patient demonstrated uniform biochemical properties. Moreover, in sCJD, four distinct biochemical PrP^Sc^ subgroups were identified that correlated with the current sCJD clinico-pathological classification. In iCJD, four similar biochemical clusters were observed, but these did not correlate to any particular *PRNP* 129 polymorphism or western blot PrP^res^ pattern. The identification of four different PrP^Sc^ biochemical subgroups in sCJD and iCJD, irrespective of the *PRNP* polymorphism at codon 129 and the PrP^res^ isoform provides an alternative biochemical definition of PrP^Sc^ diversity and new insight in the perception of Human TSE agents variability.

## Author Summary

Prion diseases are transmissible neurodegenerative disorders characterized by accumulation of an abnormal isoform (PrP^Sc^) of a host-encoded protein (PrP^C^) in affected tissues. According to the prion hypothesis, PrP^Sc^ alone constitutes the infectious agent. Sporadic Creutzfeldt-Jakob disease (sCJD) is the commonest human prion disease. Although considered as a spontaneous disorder, the clinicopathological phenotype of sCJD is variable and substantially influenced by the methionine/valine polymorphism at codon 129 of the prion protein gene (*PRNP*). Based on these clinicopathological and genetic criteria, a subclassification of sCJD has been proposed. Here, we used two new biochemical assays that identified four distinct biochemical PrP^Sc^ subgroups in a cohort of 41 sCJD cases. These subgroups correlate with the current sCJD subclassification and could therefore represent distinct prion strains. Iatrogenic CJD (iCJD) occurs following presumed accidental human-to-human sCJD transmission. Our biochemical investigations on 12 iCJD cases from different countries found the same four subgroups as in sCJD. However, in contrast to the sCJD cases, no particular correlation between the *PRNP* codon 129 polymorphism and biochemical PrP^Sc^ phenotype could be established in iCJD cases. This study provides an alternative biochemical definition of PrP^Sc^ diversity in human prion diseases and new insights into the perception of agent variability.

## Introduction

Transmissible spongiform encephalopathies (TSE) are neurodegenerative disorders affecting a large spectrum of mammalian species that share similar characteristics, including a long incubation period (which in man may be measured in decades) and a progressive clinical course resulting in death [1].

The most common form of human TSE is an idiopathic disorder named sporadic Creutzfeldt-Jakob disease (sCJD). sCJD is not a uniform disorder in terms of its clinical and neuropathological phenotype. It remains unclear whether this variability is related to variations in the causative TSE agent strains, or to the influence of the methionine/valine polymorphism at codon 129 of the *PRNP* [2],[3].

A key event in the pathogenesis of TSE is the conversion of the normal cellular prion protein (PrP^C^, which is encoded by the *PRNP* gene) into an abnormal disease-associated isoform (PrP^Sc^) in tissues of infected individuals. Conversion of PrP^C^ into PrP^Sc^ is a post-translational process involving structural modifications of the protein and resulting in a higher β-sheet content [4]. PrP^C^ is completely degraded after controlled digestion with proteinase K (PK) in the presence of detergents. PrP^Sc^ is N-terminally truncated under such conditions, resulting in a PK resistant core, termed PrP^res^ [5]. PrP^res^, also named PrP 27–30, is a disease marker for TSE and the presence of PrP^Sc^ seems to correlate with infectivity [5],[6]. According to the prion hypothesis, PrP^Sc^ is the infectious agent in TSE [7] and, in the last decades, several lines of evidence have indicated that particular biochemical properties of PrP^Sc^, such as solubility in *N*-lauroylsarcosine, PK resistance and electromobility in western blotting (WB) can be used to distinguish between different prion agents or strains [8],[9].

In sCJD, two major PrP^res^ types have been described by WB: in type 1 PrP^res^, the unglycosylated fragment is 21 kDa, while in type 2, the apparent molecular weight of this unglycosylated fragment is 19 kDa [3]. Protein N-terminal sequencing revealed that type 2 isoform derives from preferential cleavage of the protein during PK digestion at amino acid 97, while in type 1 preferential cleavage occurs at amino acid 82 [10]. sCJD cases can be subclassified according the PrP^res^ isoform and the *PRNP* codon 129 methionine (M)/valine (V) polymorphism, resulting in 6 major subypes: MM1, MM2, MV1, MV2, VV1 and VV2. Interestingly, these subtypes appear to carry distinct pathological and clinical features, [2],[3], and it has been proposed that type 1 and type 2 isoforms in sCJD might correspond to different TSE agent strains. However, the description of PrP^res^ isoforms which appear to be distinct from type 1 and type 2, and the increasing number of reports describing the coexistence of type 1 and type 2 PrP^res^ in different areas or the same area in the brain from a single sCJD patient, calls into questions the subclassification system described above in sCJD [11]–[14]. Here, in a large group of cases including 41 sCJD and 12 iCJD patients, we confirmed that type 1 and type 2 PrP^res^ can be observed as a mixture in a substantial number of patients. However, using two novel assays described here, PrP^Sc^ from these patients with mixed PrP^res^ types are homogeneous irrespective of the brain area considered. Moreover, based on these novel PrP^Sc^ biochemical properties, four distinct subgroups were observed in our cohort of sCJD patients. Similar findings were observed in iCJD cases from two countries and differing sources of infection.

## Materials and Methods

### Cases Studied

A total of 41 French cases of sCJD, each of which had frozen tissue (2–4 g) available from preferentially 5 brain regions: (occipital, temporal and frontal cortex, cerebellum and the caudate nucleus), were included in this study. All six currently defined classes of s-CJD patients (MM1-MM2-MV1-MV2-VV1-VV2) were represented in our panel (Table 1). Moreover, 12 cases of iatrogenic CJD (iCJD), linked to contamination by growth hormone (GH) or dura mater grafts, from patients originating either from United Kingdom (UK) or France, were also investigated (Table 1). None of the patients had a familial history of prion disease and, in each case, the entire *PRNP* coding sequence was analyzed, either by denaturating gradient gel electrophoresis and/or direct sequencing. All patients died from CJD during the period 1997–2004. Additionally, five cases of Alzheimer's disease were included as non-CJD controls.

**Table 1:** Abnormal PrP Properties as Assessed by Western Blot, PK Digestion ELISA and Strain Typing ELISA in 41 French Sporadic CJD Patients and 12 Iatrogenic CJD Patients Originating either from France or the United Kingdom

Codon 129	3F4 Type	Number	Etiology	Sha 31 PrP^res^ WB type					20% Signal PK Concentration (µg/ml)^b^		Ratio in CEA Strain Typing Test^c^		PrP^Sc^ Groups
				Front Cort.	Caud Nucl.	Cerebel	Occipt. Cort.	Temp Cort	Mean–SD	Min–Max	Mean–SD	Min–Max	
MM	1	*n* = 11	Sp	1	1	1	1	1	—	>500	0.20–0.02	0.17–0.23	Group 1
MM	1	*n* = 1	Sp	1+2	—	1	—	—	—				
MM	1	*n* = 1	Sp	1	—	1+2	—	—	—				
MV	1	*n* = 8	Sp	1	1	1	1	1	—	>500	0.22–0.05	0.18–0.32	Group 1
VV	2	*n* = 8	Sp	1	2	2	2	2	171.6–21.4	139–193	1.76–0.2	1.45–2.09	Group 2
				2	1	2	2	2					
				1+2	1	2	2	2					
				2	2	2	2	2					
				1+2	1+2	2	1+2	1+2					
				2	2	2	2	2					
				2	2	2	2	2					
				1+2	2	1	1+2	2					
MV	2	*n* = 8	Sp	2	1	2	1+2	2	164.3–13.7	149–187	1.63–0.34	1.23–2.05	Group 2
				2	2	2	2	2					
				1+2	1	1+2	2	2					
				1+2	1+2	2	2	1+2					
				2	2	2	2	2					
				2	1+2	1	2	1+2					
				2	1+2	1	2	2					
				2	1	1+2	2	1+2					
VV	1	*n* = 1	Sp	1	1	1	1	1	268.3–17.4	249–284	0.58–0.02	0.56–0.60	Group 3
MM	2	*n* = 3	Sp	2	2	2	2	2	98.3–12.2	87.4–117.2	3.25–0.35	3.0–3.78	Group 4
MM	1	*n* = 2	DM^a^	1	—	—	—	—	—	>500	—	0.22–0.25	Group 1
MM	1	*n* = 3	GH	1	1	1	1	1	282.1–28.6	259–301	0.64–0.06	0.57–0.71	Group 3
MV	1	*n* = 2	GH	1	1	—	—	1	276–15.8	263–289	—	0.49–0.62	Group 3
MV	1	*n* = 1	GH	1	1	—	—	1	162–4.3	153–176	—	1.28–1.84	Group 2
MV	2	*n* = 1	GH^a^	2	1+2	1+2	—	2	174.4–13.3	160–192	—	1.04–1.35	Group 2
VV	1	*n* = 1	DM	1	1	1	1	1	111.3–2.4	109–113	—	3.0–4.75	Group 4
VV	2	*n* = 2	GH^a^	2	—	—	—	—	—	165–177	—	1.38–1.45	Group 2

Sp, Sporadic; DM, iatrogenic cases linked to dura mater grafts; GH, iatrogenic cases linked to growth hormone treatment.

a Patients originating from the United Kingdom.

b Express the PK concentration for which, when increasing PK concentration, the ELISA PrP^Sc^ signal reach an arbitrary cut-off value set at 20% of the signal observed with a PK concentration of 50 µg/ml.

c Express the ratio of ELISA signal obtained (A/A′) after PrP^sc^ PK digestion differential PK digestions in a non-perturbing detergent mixture (A), and a denaturing (SDS) detergent mixture (A′).

In all cases, informed consent for research was obtained and the material used had appropriate ethical approval for use in this project.

### Tissue Homogenate Preparation

For each sample, a 20% brain homogenate (weight/volume) in 5% glucose was prepared using a high-speed homogenizer (TeSeE Precess 48 system). The homogenates were then filtered through a 20 Gauge needle before storage at −80°C.

### Western Blot PrP^sc^ Banding Pattern

Various factors have been reported to influence the results of PrP^res^ analysis by WB, including tissue pH and the effect of Cu^2+^ ions [15]–[17]. In order to limit these factors, each homogenate was diluted a 100-fold in a single non-CJD control brain homogenate prior to further investigation.

A WB kit (TeSeE WB kit Bio-Rad) was used following the manufacturer's recommendations.

Three different monoclonal PrP-specific antibodies were used for PrP detection: Sha31 (1 µg/ml) [18], 8G8 (4 µg/ml) [19] and 12B2 (4 µg/ml) [20], which recognized the amino acid sequences YEDRYYRE (145–152), SQWNKPSK (97–104) and WGQGG (89–93) respectively. After incubation with goat anti-mouse IgG antibody conjugated to horseradish peroxidase, signal was visualized using the ECL western blotting detection system by enhanced chemiluminescent reaction (ECL, Amersham). Molecular weights were determined with a standard protein preparation (MagicMark, Invitrogen).

### PrP^Sc^ Resistance ELISA

PrP^Sc^ detection was carried out using sandwich ELISA test (TeSeE CJD, Bio-Rad) used following the manufacturer's recommendations. The assay protocol includes a preliminary purification of the PrP^Sc^ (TeSeE purification kit) consisting in (i) digestion of PrP^C^ with PK, (ii) precipitation of PrP^Sc^ by buffer B and centrifugation, (iii) denaturation of PrP^Sc^ in buffer C at 100°C, before immuno-enzymatic detection. In this ELISA, the capture antibody 3B5 recognizes the octarepeat region of PrP [19], while the detection antibody 12F10 binds to the core part of the protein [18].

PK resistance of the PrP^Sc^ portion recognized in the ELISA test was determined by measurement of the ELISA specific signal recovered from a series of homogenate aliquots digested with different concentrations of PK in buffer A′ reagent (TeSeE Sheep/Goat purification kit). Each sample was first diluted in normal brain homogenate (between 100- and 10,000-fold) until obtaining a signal between 1.5 and 2 absorbance units after digestion with 50 µg/ml of PK. Triplicate of equilibrated samples were then submitted to a PK digestion with concentrations ranging from 50 to 500 µg/ml, before PrP^Sc^ precipitation and ELISA detection. Results were expressed as the percentage of residual signal when compared to the 50 µg/ml PK digestion (lowest PK concentration). In each assay, two standardized controls (scrapie and BSE from sheep) were used as an internal standard. About 20% of samples were randomly selected and submitted to two independent tests separated in time as to assess inter-assay variation.

### “Strain Typing” ELISA

The ELISA test used in this study was adapted from the Bio-Rad TeSeE test, validated at CEA for EU strain typing studies in ruminants and designed to distinguish BSE in sheep from scrapie. The principle was to measure conformational variations in PrP^Sc^ by applying two differential PK digestions under the modification of detergent conditions (SDS sensitivity). For each sample, PK digestion was performed under two conditions: (i) two aliquots of 250 µl of 20% homogenate were mixed either with 250 µl of A reagent (TeSeE purification kit) containing 20 µg of PK, (ii) with 250 µl of A′ reagent (N-lauroylsarcosine sodium salt 5% (W/V), sodium dodecyl sulfate 5% (W/V) containing 55 µg of PK, All the tubes were then mixed by inversion 10 times and incubated at 37°C (in a water bath) for exactly 15 min. Subsequently, 250 µl of reagent B (Bio-Rad purification kit)/PMSF (final concentration 4 mM) were added, mixed and the tubes were centrifuged for 5 minutes at 20,000 *g* at 20°C. Supernatants were discarded and tubes dried by inversion onto an absorbent paper for 5 min. Each pellet was denatured for 5 min at 100°C with 25 µl of C reagent (Bio-Rad purification kit). The samples were diluted in 250 µL of R6 buffer containing 4 mM of serine protease inhibitor AEBSF (4-2-aminoethyl benzenesulfonyl fluoride hydrochloride), and, if desired, further serially diluted in R6 buffer. ELISA plates were then incubated for two hours at room temperature and, after three washes, antibody detection (TeSeE CJD, Bio-Rad) was added for two hours at 4°C. The ratio of the absorbance obtained in the two conditions (A/A′) was calculated using appropriate dilutions providing absorbance measurements ranging from 0.5 to 2.5 absorbance units in A conditions. For each plate, the same three control samples (one MM1, one VV2 and one MM GH) were included. To avoid inter-assay variations, final results were expressed as a normalized ratio established by dividing the ratio obtained for the analyzed sample by the one obtained for a VV2 sample selected as standard.

## Results

### Intra-Individual Variability of WB Banding Pattern

**Figure 1:**
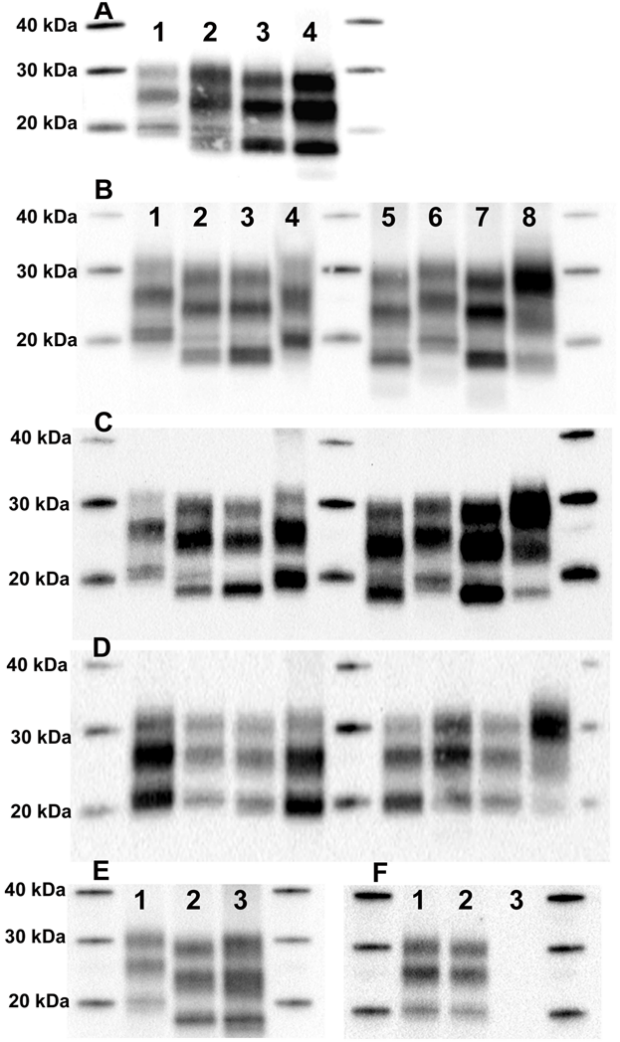
PrP^res^ Profiles in sCJD Patients. (A) PrP^res^ from cerebellum (lane 1), caudate nucleus (lane 2), frontal cortex (lane 3) and occipital cortex (lane 4) of a single MV patient was extracted and submitted to WB. Detection with antibody Sha31 reveals different PrP^res^ profiles. (B–D) PrP^res^ in cerebellum from seven different patients using Sha31 mAb (B), 8G8 mAb (C), and 12B2 mAb (D). Because of their epitope, Sha31 and 8G8 can detect both type 1 and type 2 PrP^res^ while 12B2 detects type 1 only. Lane 1, MM patient; lane 2, MV patient; lane 3, VV patient; lane 4, MV patient; lane 5, MV patient; lane 6, VV patient; lane 7, MM patient; line 8, sheep BSE control. (E, F) PrP^res^ profile in temporal cortex from three MV patients revealed by Sha31 mAb (E) or 12B2 mAb (F).

For each sCJD case, PrP^res^ profile was determined from five brain areas using both Sha31 and 8G8 antibodies. A single PrP^res^ type (type 1 or type 2) was observed in investigated brain areas of most of the MM1 (n = 11), and all the areas from MV1 (n = 8), VV1 (n = 1) and MM2 (n = 3) sCJD cases of our panel. However, in several cases initially classified as MM1 (n = 2), and in a majority of VV2 (n = 5) or MV2 (n = 6) cases, some brain areas harboured mixed electrophoretic pattern characterized by two distinct bands at 19 and 21 kDa, indicating the coexistence of PrP^res^ type 1 and type 2 (Table 1 and Figure 1A). Moreover, in individual patients some brain areas were found to be type 1, while another area could be found to be type 2 (Table 1 and Figure 1A). Since both Sha31 and 8G8 gave similar results this phenomenon cannot be attributed to some antibody peculiarity in PrP recognition (Figure 1B and 1C).

Antibody 12B2 is specific for the amino acid sequence 89–93 that is located N-terminally of the type 2 cleavage site (amino acid 97). In principle, this antibody is unable to recognize type 2 PrP^res^. Systematic western blotting with 12B2 consistently demonstrated the presence of the 21 kDa band, characteristic for type 1 PrP^res^, in nearly all type 2 classified samples, regardless of the *PRNP* codon 129 polymorphism (Figure 1D). In a limited number of type 2 samples, 12B2 failed to detect a type 1 band (Figure 1E and 1F, lane 2,3).

**Figure 2:**
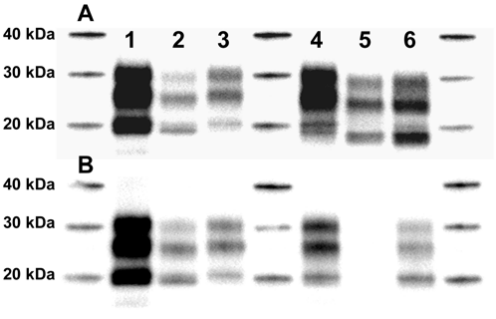
PrP^res^ Profiles in iCJD Patients. PrP^res^ (frontal cortex) was revealed in WB by antibodies Sha31 (A) and 12B2 (B). Lane 1, MM UK dura mater patient; lane 2, VV French dura mater patient; lane 3, MM GH French patient; lane 4, French MV GH patient; lane 5, MV UK GH patient; lane 6, VV UK GH patient.

Using Sha31 or 8G8, mixed type 1/type 2 PrP^res^ profiles were observed in several iCJD cases (Figure 2A), regardless of their national origin or mode of infection. In most (but not all) samples initially classified as type 2, the 12B2 antibody revealed the presence of a 21 kDa band, characteristic of type 1 PrP^res^ (Figure 2B).

Together these findings point to the existence of variable amounts of type 1 PrP^res^ molecules in all or nearly all type 2 classified patients (Table 1).

### All Brain Areas from a Single Patient Have Similar PrP^Sc^ Features

**Figure 3:**
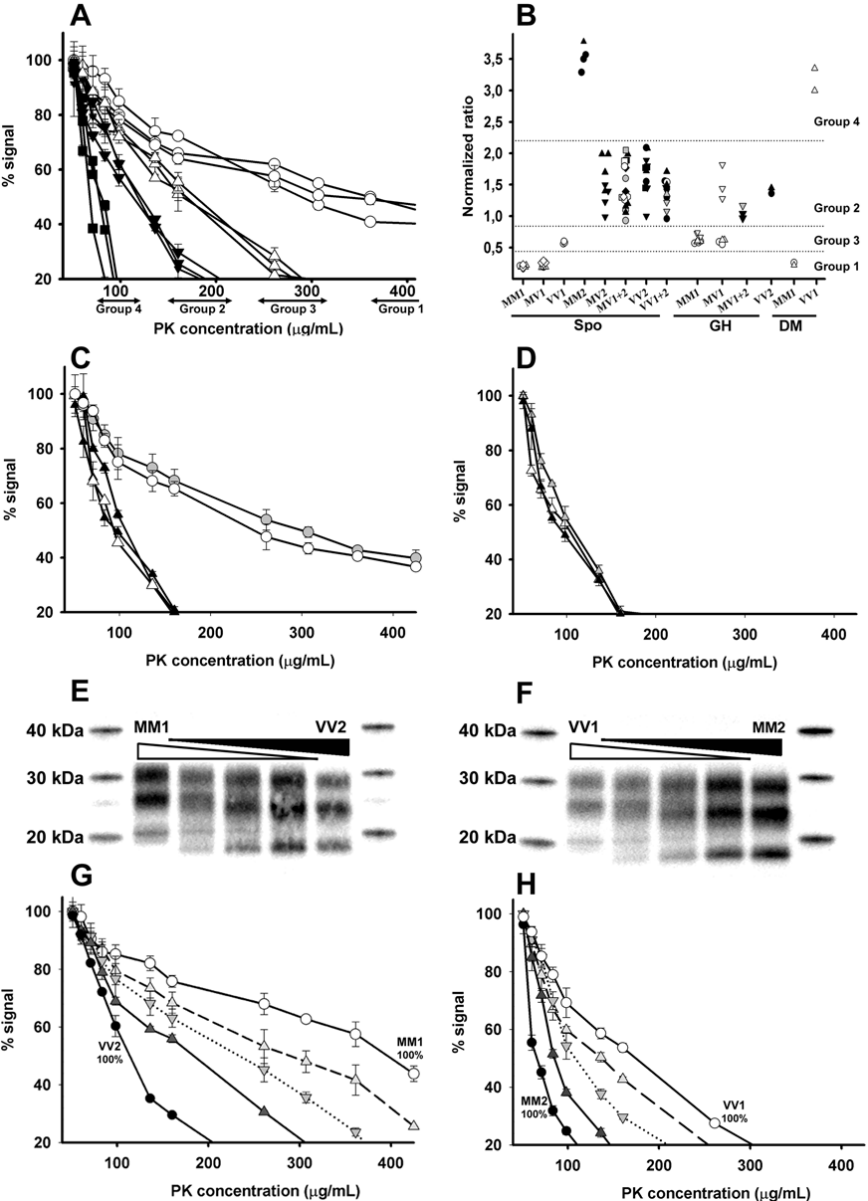
PrPsc PK Resistance and Molecular Strain Variations in sCJD and iCJD Brain Samples. Each investigated brain sample was initially characterized by WB using antibody Sha31. Symbol patterns represent type 1 in white, type 1+2 in grey and type 2 in black. (A) Results from PK resistance ELISA carried out on three different brain areas (cerebellum, caudate nucleus and temporal cortex) from a MM1 (open circles), a VV1 (open triangles), a VV2 (inverted filled triangle) and a MM2 (filled squares) sCJD patient. Values obtained are expressed as percentage of signal obtained with the lowest PK concentration (50 µg/mL). (B) Results from CEA strain typing ELISA (one symbol per patient—3 to 5 different areas by patients). PrP^Sc^ signal intensity was measured after PK digestion into two different detergent solutions. Normalized A/A′ ratio was calculated for each sample (see Methods section). MM1 and MV1 had a low ratio indicating an absence of alteration of PrP^sc^ PK sensitivity linked to the modification detergent digestion conditions. This ratio was higher in MV2, VV2, MV1+2, and VV1+2, while, in the unique VV1 case, an intermediate ratio was observed. In MM2 patients, the huge ratio indicated a strong increase in PK sensitivity by modification of detergent conditions. (C, D) PK resistance assay in three areas from a (C) VV (triangles) or MM (circles) patient and in (D) a MV (triangles) patient harbouring distinct PrP^Sc^ WB type in their different brain areas. Artificial mixtures of MM2/VV1 or MM1/VV2 samples were prepared. All homogenates were first equilibrated by dilution into negative brain homogenate to obtain an equal PrP^Sc^ signal in ELISA. (E, F) Mixtures were then tested by Western Blot (200 µg PK digestion—Sha31 anti PrP antibody). (E) Lane 1: MM1 100%; Lane 2: MM1 75%/VV2 25%; Lane 3: MM1 50%/VV2 50%; Lane 4: MM1 25%/VV2 75%; Lane 5: VV2 100%. (F) Lane 1: VV1 100%; Lane 2: VV1 75%/MM2 25%; Lane 3: VV1 50%/MM2 50%; Lane 4: VV1 25%/MM2 75%; Lane 5: MM2 100%. (G, H) Same mixtures were tested in the PK resistance ELISA assay. (G) VV2 100% (filled circles), VV2 75%/MM1 25% (filled triangles), VV2 50%/MM1 50% (filled inverted triangles), VV2 25%/MM1 75% (open triangles), MM1 100% (open circles). (H) MM2 100% (filled circles), MM2 75%/VV1 25% (filled triangles), MM2 50%/VV1 50% (filled inverted triangles), MM2 25%/VV1 75% (open triangles), VV1 100% (open circles).

In sCJD and iCJD patients who harboured a single WB PrP^Sc^ type in the different brain areas, as assessed by Sha31, a single ELISA PK resistance profile (Table 1 and Figure 3A) and a comparable ratio in strain typing assay (Table 1 and Figure 3B) were observed in all brain areas. Surprisingly, in each patient harbouring both type 1 and 2 PrP^res^, either in the same or in different brain areas, a single ELISA PK digestion profile (Table 1 and Figure 3C and 3D) and a comparable signal ratio in strain typing assay (Table 1 and Figure 3B) was also observed, irrespective of region assayed.

MM1 and VV2 samples but also MM2 and VV1 samples, which harboured similar apparent PrP^Sc^ content (as assessed by ELISA) were artificially mixed in different proportions. Using WB, a mixed type 1+2 profile could, or could not, be observed depending on the mixture proportions (Figure 3E and 3F). Both PrP^Sc^ resistance ELISA assay (Figure 3G and 3H) and strain typing ELISA (not shown) were able to discriminate the different mixtures from the original isolates and from each other. These results clearly demonstrate that the uniformity of PrP^Sc^ biochemical properties, as demonstrated by both PrP^Sc^ resistance ELISA and strain typing ELISA, in patients harbouring different PrP^res^ isoforms cannot be attributed to a lack of discriminative power of these techniques.

Together, these data strongly indicate that, despite possible variations in PrP^res^ type on WB analysis, patients with either sCJD or iCJD appear to harbour a single PrP^Sc^ isoform in their brain.

### Four Distinct PrP^Sc^ Biochemical Signatures Are Observed in iCJD and sCJD Patients

According to the results from PrP^Sc^ PK resistance assay and strain typing ELISAs, sCJD patients could be split into four groups (Table 1, and Figure 3A and 3B). The first group was characterized by a strong PK resistance (Figure 3A) and a low ratio in strain typing assay (Figure 3B). Group 1 could be readily differentiated from Group 2 which showed a higher sensitivity regarding PK digestion, as well as an increased signal ratio in strain typing assay, when compared to Group 1. Two other PrP^Sc^ groups were also observed. Group 3 harboured an intermediate PK lability in the PrP^Sc^ resistance ELISA and ratio in the strain typing ELISA, when compared to Group 1 and 2. Group 4 had a very high PK-sensitivity and ratio in the strain typing ELISA. No overlapping in PK resistance profile or ratio value in strain typing assay were observed between the four determined groups (Table 1).

Group1 was composed of sCJD MM and MV patients, harbouring predominantly type 1 PrP^res^ while Group 2 consisted in VV and MV patients harbouring predominantly type 2 or type 1+2 PrP^res^. Groups 3 and 4 were respectively composed with VV1 and MM2 patients from our sCJD panel.

**Figure 4:**
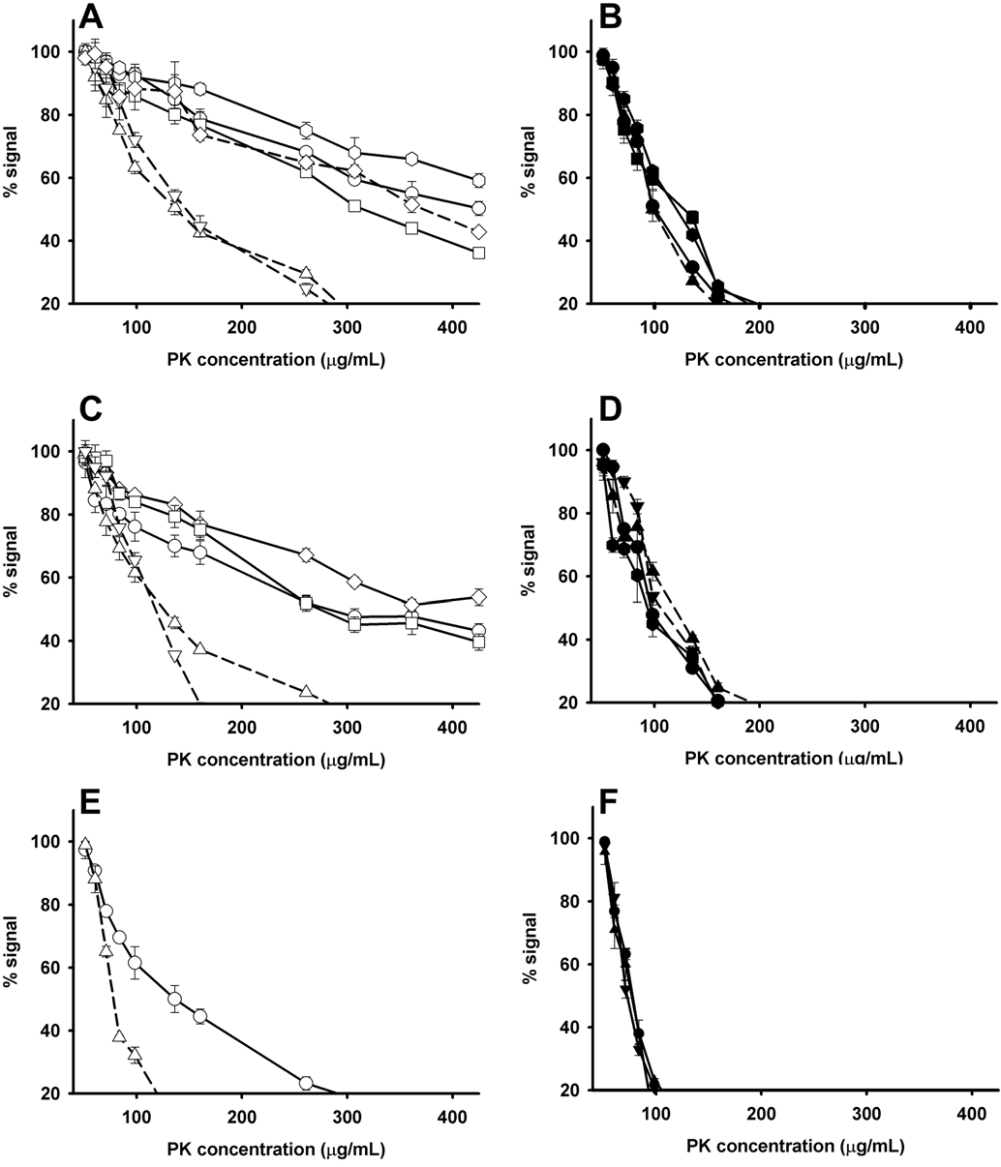
PK Resistance ELISA in Frontal Cortex from sCJD and French or UK iCJD (Growth Hormone and Dura Mater Cases). sCJD (solid line) and i-CJD (dashed line) frontal cortex samples were investigated by PK resistance assay. (A) MM1 sCJD cases (open circles, hexagons and squares), MM1 iCJD UK dura mater cases (open diamonds), MM1 French GH cases (open triangles). (B) MV2 sCJD cases (filled circles, hexagons and squares) and MV2 UK GH case (filled triangles). (C) MV1 sCJD (open circles, diamonds and squares) and MV1 French GH (open triangles) cases. (D) VV2 sCJD (filled circles and hexagons) and VV2 UK GH (filled triangles) cases. (E) VV1 sCJD case (open circles) and VV1 dura mater French case (open triangles). (F) MM2 sCJD cases (filled circles and triangles).

Striking differences were observed in the PrP^Sc^ properties between the different iCJD cases and all four groups relying on PrP^Sc^ signatures observed in sCJD cases were identified (Table 1, and Figures 3B and 4).

As it might have been expected from sCJD cases observations, Group 1 PrP^Sc^ properties was identified in MM1 UK dura mater graft patients (n = 2) (Figure 4A) while Group 2 PrP^Sc^ features were observed in UK VV2 (n = 2) (Figure 4D) and MV2 (n = 1) (Figure 4B) GH patients. Surprisingly, a typical Group 2 PrP^Sc^ signature was also observed in one out of the three MV1 French GH patients (type 1 in all brain areas). Meanwhile, all investigated MM1 and two out of the three MV1 French GH cases (Figure 4A) harboured identical PrP^Sc^ properties than Group 3 sCJD (Figure 4E). Finally, a Group 4 sCJD PrP^Sc^ signature (Figure 4F) was observed, using both PrP^Sc^ resistance ELISA (Figure 4E) and strain typing ELISA (Figure 3B), in a French dura mater VV1 case (n = 1), which harboured a type 1 PrP^res^ WB profile in every investigated area.

Taken together, these observations support the concept that, in iCJD patients, variability in the PrP^Sc^ biochemical properties is not related to the route of infection or the *PRNP* codon 129 genotype. It also indirectly suggests that the range of different PrP^Sc^ properties observed in iCJD might be related to those in the source of infection (likely to have been a sCJD case).

## Discussion

### Coexistence of Different PrP^res^ Types in the Same Subject

In this study, detection, by WB, of the coexistence of two PrP^res^ types in about 30% (13/41) of cases is consistent with already published data [12],[14]. This observation could suggest the existence in brain from a single patient of different abnormal PrP species. Although two main PK cleavage sites are associated with PrP^res^ type 1 and type 2 (respectively amino acid 82 and 97), N-terminal sequencing revealed in all investigated cases the presence of a whole spectrum of overlapping cleavage sites. Moreover in a part of investigated cases this technique demonstrated the presence (i) of variable but consistent level of type 1 PrP^res^ in patients classified type 2 using WB and (ii) in some patient classified type 1, of low amount of type 2 PrP^res^ [10]. These observations could suggest that, rather than a pure type 1 or type 2 PrP^res^, PK digestion of a PrP^Sc^ specific conformer generate variable mixture of PrP^res^ fragments (with presence of dominant or sub dominant type 1 or type 2 PrP^res^), which WB usually failed to reveal accurately because its intrinsic technical limits [14]. Antibodies either harbouring higher affinity to PrP (like Sha31) [18] or probing specifically type 1 PrP^res^ (like 12B2) [20], now allow a better perception of such mixture. However, investigations carried out using artificial mixture of type 1 and type 2 brain homogenate, even using high affinity anti-PrP antibodies, clearly indicate the current limits of WB discriminative power [14]. Together, these data suggest that WB analysis of PrP^res^ on its own could be misleading for adequate discrimination between PrP^Sc^ variants in CJD.

Both PrP^Sc^ PK resistance ELISA and strain typing ELISA are based on the characterization the N terminal part of the PrP^Sc^ PK digestion either by increasing PK amount or modifying detergent conditions. While WB profile could be compared to a snapshot picture of PrP^res^ fragments generated by PK digestion process, these assays reflect the dynamics of the PK cleavage rather than its final result (different forms of PrP^res^). Consequently they could provide different but also more accurate perception of the PrP^Sc^ conformers.

Our findings from the PrP^Sc^ capture immunoassays clearly indicate that in a single patient, irrespective of brain area, sCJD associated PrP^Sc^ displays uniform biochemical properties, regardless of the regional variation of type 1 and type 2 isoforms determined by WB. Such findings support the idea of the presence of a specific TSE agent in each brain and the accumulation of a single associated PrP^Sc^ conformer.

### sCJD Classification

Because the limited size of our cohort of cases, an in depth comparison between the PrP^Sc^ signature (as established in this study) and the Parchi classification system is not possible.

However, despite this limitation, two major groups were identified in our panel according to the PrP^Sc^ properties. The first major group was constituted with patients harbouring a highly PK resistant PrP^Sc^ (MM1 and MV1 patients). The second group included patients harboring a PK labile PrP^Sc^ (VV2 and MV2 patients). Using both lesion profile and clinical parameters [2], two major forms of sCJD are commonly recognized. The first sCJD form, named “classical”, is characterized by a “rapid evolution” (usually around 4 months), and affects most of the MM1 and MV1 patients. The second sCJD form, named “atypical”, affects VV2 and MV2 with a longer symptomatic evolution (usually longer than 6 months) and a late dementia. Despite inter-individual variations, sCJD Groups 1 and 2, as we defined them on biochemical criteria were consistent with this classification.

Both VV1 and MM2 sCJD cases are extremely rare; they respectively represent 1% and 4% of the identified sCJD cases. According to the literature, these patients have clinical features and lesion profiles that are very different from other sCJD patients [2]. However, in our study as in previously published studies, WB did not identify any distinct biochemical difference from other type 1 and type 2 cases. In contrast, both the strain typing ELISA and PrP^Sc^ resistance assays clearly differentiated these cases from Group 1 and Group 2 cases. This finding, which is consistent with clinico/pathological observations carried out in patients, could indicate that there are indeed differences in PrP^Sc^ that distinguish these VV1 and MM2 cases from other sCJD groups.

### Prion Strains and PrP^Sc^ Phenotype

Although prion strains can only be identified definitively by bioassay, molecular in vitro tools to characterize PrP^Sc^ are more and more widely used for the rapid identification of particular agents, such as BSE in cattle, sheep, rodent and humans (vCJD) [20],[21]. This has come to be termed “molecular strain typing” and although widely employed, the exact relationship between PrP^Sc^ biochemistry and the biological properties of the agents responsible remain to be determined. In sCJD, the presence of four distinct PrP^Sc^ biochemical forms apparently correlated to clinico-pathological phenotypes as defined by Parchi et al. [2] could be an indication of the involvement of different TSE agents.

iCJD cases are a consequence of accidental human to human TSE transmission, most likely representing transmission of sCJD. The identification in iCJD cases of the four PrP^Sc^ signatures identified in sCJD is consistent with the existence of distinct prions associated with these biochemical forms.

Three examples of human-to-human transmission of variant CJD through blood transfusion have now been identified. While all blood donors were MM at codon 129 *PRNP*, the recipients had either a MM (n = 2) or a MV genotype (n = 1). Despite this genotype difference there appears to have been conservation of the disease phenotype and PrP^res^ type in all “secondary” vCJD cases [22]–[25]. These observations could suggest that in case of inter-human transmission, difference in donor/recipient genotype could result in un-altered abnormal PrP signature.

Our identification of MM GH iCJD cases harbouring similar PrP^Sc^ signature as a VV1 sCJD case or of a VV dura mater iCJD case similar to MM2 sCJD might indicate preservation of a specific PrP^Sc^ biochemical signature after human to human transmission between individuals of different codon 129 genotypes.

Treatment with extracts of GH contaminated by CJD has lead to a high number of iCJD cases in France and the UK. The codon 129 genotypes of the affected individuals in the two countries differ, with the French cohort predominantly MM and MV and the British cohort MV and VV [26]. In the absence of any clear explanation for this finding, it was suggested that it might be due to contamination of different batches of GH with different prion strains from individuals of differing *PRNP* codon 129 genotypes. Our identification of different biochemical forms of PrP^Sc^ in GH French patients and in UK patients is consistent with this hypothesis. The variability observed within the French GH cases could signify involvement of different prion strains, consistent with multiple contaminated GH batches in the French epidemic.

### Conclusion

The identification in this study of different PrP^Sc^ species in CJD patients with the same *PRNP* polymorphism at codon 129 and WB PrP^res^ profile offers a new perspective on our understanding of the relationship between PrP biochemistry, prion disease phenotype and agent strain. We highlight two novel approaches to analysing PrP^Sc^ in sCJD and iCJD and offer evidence that these analyses provide potentially-strain associated information, which appears to be lacking from the conventional WB assay.
